# Consumption of sugar-sweetened beverages and artificially sweetened beverages from childhood to adulthood in relation to socioeconomic status – 15 years follow-up in Norway

**DOI:** 10.1186/s12966-018-0646-8

**Published:** 2018-01-17

**Authors:** Kathrine Bolt-Evensen, Frøydis N. Vik, Tonje Holte Stea, Knut-Inge Klepp, Elling Bere

**Affiliations:** 1 Department of Public Health, Sport and Nutrition, Faculty of Health and Sport Sciences, University of Agder, PO. Box 422, 4604 Kristiansand, Norway; 2 Department of Nutrition, Faculty of Medicine, University of Oslo, Oslo, Norway

**Keywords:** Sugar-sweetened beverages, Artificially sweetened beverages, Socioeconomic status, Time trends, Longitudinal

## Abstract

**Background:**

In Norway, social inequalities in health and health-related behaviors have been reported despite the well-developed welfare state. The objective of the present study was to analyze; (i) the development in frequency of consumption of sugar-sweetened beverages (SSB) and artificially sweetened beverages (ASB) from childhood to adulthood; (ii) socioeconomic inequalities in the consumption of SSB and ASB using different indicators of socioeconomic status (SES); (iii) time trends in potential disparities in SSB and ASB consumption among different socioeconomic groups to assess the development in socioeconomic inequality from childhood to adulthood.

**Methods:**

This study uses data from the Fruits and Vegetables Make the Marks (FVMM) longitudinal cohort, including participants (*n* = 437) from 20 random schools from two Norwegian counties. Data from the first survey in 2001 (mean age 11.8) and follow-up surveys in 2005 (mean age 15.5) and 2016 (mean age 26.5) were used. Consumption of SSB and ASB were measured using a food frequency questionnaire, which the participants completed at school in 2001 and 2005, and online in 2016. Various indicators of SES were included; in 2001, parental education and income were measured, in 2005, participants’ educational intentions in adolescence were measured, and in 2016, participants’ own education and income were measured. The main analyses conducted were linear mixed effects analysis of the repeated measures.

**Results:**

Between 2001 and 2016, a decrease in frequency of consumption of SSB (2.8 *v* 1.3 times/week; *p* = < 0.001) and an increase in frequency of consumption of ASB (1.1 *v* 1.6 times/week; *p* = 0.002) were observed. Participants with a higher educational level in adulthood and higher educational intentions in adolescence had a significantly lower frequency of consumption of SSB at all time points (2001, 2005 and 2016). No significant widening (or narrowing) of inequalities were observed from childhood to adulthood.

**Conclusions:**

A decrease in consumption of SSB and an increase in consumption of ASB from childhood to adulthood were found. Participants with high SES consumed in general less SSB (but not ASB), however, results varied depending on SES indicator used. The established inequalities persisted from childhood to adulthood.

## Background

A number of studies have reported socioeconomic inequalities in health and health-related behaviors in several European countries [1, 2]. This is of concern also in welfare states traditionally marked by commitment to social equality, such as the Nordic countries [3]. People with low socioeconomic status (SES) are at risk of consuming poorer diets, with lower fruit and vegetable consumption and higher intake of unhealthy snacks, fast food and sugar-sweetened beverages (SSB) [4]. Further, people higher in the socioeconomic hierarchy tend to live longer and have reduced prevalence of most types of health problems [5]. In Norway, national health authorities have invested considerable effort to reduce socioeconomic inequalities in health and health-related behaviors [6]. Despite these efforts, long-term trends of social inequalities in mortality show that Norway have larger inequalities in mortality than other European countries [7].

As a result of the increased focus on reducing intake of added sugar and consumption of SSB in the population, several environmental and structural initiatives have been made in Norway. From 2007 to 2011 the national health authorities had a specific goal to reduce the proportion of people with a daily consumption of SSB by 20% [8], and from 2017 to 2021 to reduce the proportion of adolescents who drink SSB five times a week or more by 50% [9]. The Norwegian state has introduced a tax on non-alcoholic beverages containing added sugar or artificial sweeteners that was increased from 1.68 Norwegian kroner (NOK) per liter to NOK 2.76 per liter between 2008 and 2010 and further to NOK 3.27 per liter in 2016 [10–12]. Further, the Norwegian health authorities have supported the WHO initiative to reduce marketing of unhealthy foods and beverages aimed at children and young people [13, 14]. In addition, it is specified in recommendations related to school meals that schools should prevent access to soft drinks [15]. In the Norwegian food-based dietary guidelines, two out of twelve guidelines concern beverage intake; one regards the avoidance of beverages and foods rich in added sugar on an everyday basis and one recommends drinking water when thirsty [16].

Trends in sales of SSB based on the Norwegian food consumption surveys show that sales of SSB are more than tenfold since the 1950s [17]. The sale of SSB reached its highest in 1997 and has later decreased over time from 60 l/person per year (l/person) in 2007 to 55 l/person per year in 2015 [17]. National representative dietary surveys have revealed that one of the most important health related-problems in the diet of children, adolescents and adults still is a high intake of added sugar [18, 19]. Further, the major source of added sugar in the diet of Norwegians was SSB [18, 19]. A Norwegian longitudinal study with the objective to investigate the tracking of SSB intake from age 15 to 33 years showed that intake of SSB from early adulthood at 25 years of age into later adulthood at 33 years of age was relatively stable, while the stability in SSB intake from 15 years of age into later adulthood was low [20].

Samdal and colleagues have reported a social gradient in children’s consumption of SSB where 20% of children with low SES consumed SSB five times a week or more often compared to 13% of children with middle SES and 10% of children with low SES [21]. Further, Bere and colleagues have reported that adolescents planning to attend college or university have lower odds of drinking SSB compared to those not planning to attend college or university [22]. In addition, beverage consumption patterns among Norwegian adults show that those with higher education have decreased odds of being consumers of SSB [23].

Internationally, a significantly increased consumption of artificially sweetened beverages (ASB) has been observed in recent years [24]. Trends in the consumption of ASB based on the Norwegian food consumption surveys show that sales of ASB has increased over time but has stagnated in recent years [17]. Several studies report that the consumption of ASB is lower than the intake of SSB, ASB intake appears to increase with age and is more common among girls than boys, and among women than men, and that people trying to lose weight report a higher intake of ASB than those not trying to lose weight [23, 25]. In addition, ASB consumers are more likely to be college educated and have higher household incomes [26].

Within the last decade, research has moved from describing the nature of the relationship between socioeconomic inequalities and health related behavior towards understanding the observed variations [27]. Few studies assess how consumption of SSB and ASB in relation to SES change across time. The present study fills a gap in the literature by exploring whether the association between SES and consumption of SSB and ASB change from childhood to adulthood. This is especially interesting regarding the large focus on reducing inequality in health and the structural initiatives conducted over the last two decades in Norway to reduce SSB.

Thus, the objective of the present study was to analyze; (i) the development in consumption of SSB and ASB from childhood to adulthood; (ii) socioeconomic inequalities in the consumption of SSB and ASB using different indicators of SES; (iii) time trends in potential disparities in SSB and ASB consumption among different socioeconomic groups to assess the development in socioeconomic inequality from childhood to adulthood.

## Methods

### Study design and sample

The present study is part of the Fruits and Vegetables Make the Marks (FVMM) project [28–34]. The project started in 2001 and included 38 schools from Hedmark and Telemark counties in Norway. Hedmark and Telemark are rural counties mainly consisting of smaller towns and villages. Small schools with fewer than 10 pupils per grade level were excluded from the sampling frame. Among the remaining primary schools in these two counties, 48 (24 per county) were selected randomly and invited to participate, and 19 schools from each county agreed to participate. All 6th and 7th graders in these 38 schools were invited to take part in the baseline survey. Further, 18 schools (9 in each county) were randomly selected for intervention in the school year of 2001/02. One of the evaluated interventions consisted of free participation in the Norwegian School Fruit Program, and the other of a fruit and vegetable classroom curriculum including parental involvement. Informed consent was sought from both parents and children prior to the study.

At the first survey in September in 2001, a total of 1950 (out of 2287 eligible, 85%) pupils completed a questionnaire (validated for fruit and vegetable intake) at school under supervision of trained project workers, and brought home a parent questionnaire to be completed by one of their parents. Fifty-nine (3%) children-parents refused to participate, one class (27 children, 1%) was not able to carry out the survey, and 251 children (11%) did not attend this specific school lesson and were not re-contacted. Age or date of birth were not recorded, but based on available data from similar surveys; the average age of the baseline sample was estimated to be 11.8 years. Overall, 1647 parents (84% of the participating pupils) completed the parent questionnaires; 84% of these were mothers/female guardians. The average age of the parents was 40.0 years.

The initial FVMM project included two further surveys (May 2002 (*n* = 1794) and May 2003 (*n* = 1032)). Later, we were allowed to contact the study sample again, informing the parents but without parental consent for participation. In May 2005 (*n* = 1601), a fourth survey was carried out at a time where the study population was in ninth and tenth grade in 33 different secondary elementary schools (mean age 15.5 years). A fifth survey was conducted in September 2009 (*n* = 320) at a time where most participants had finished high school. In 2016, the sixth survey was conducted (*n* = 982, mean age 26.5 years). Data from the baseline survey in 2001 and follow-up surveys completed in 2005 and 2016 were used for the analyses in this longitudinal cohort study. These time-points were included to ensure data from childhood, adolescence and early adulthood. Participants from the 20 control schools who had completed both the baseline survey in 2001 and follow-up survey in 2016 were included in the study (*n* = 437, 49% of control children from cohort).

In 2001 and 2005, the questionnaire surveys were completed by the pupils in the classroom under supervision of project workers on weekdays, Tuesday to Friday, during one class of 45 min. A trained project worker was present for questions and guidance of the participants. In addition, participating pupils brought home a parent questionnaire to be completed by either of their parents at the baseline survey of the project in 2001. In 2016, baseline participants were contacted mainly through Facebook, and occasionally by phone. When using the Facebook search tool to locate participants, a research profile was created for each of the four project workers, using their contact details with a picture of the University of Agder as the profile picture. Using these profiles, the participant’s name, age, elementary school, geographic location and friend list were used to search for potential cohort participants. Verified participants received a personal message with information about the project. Further, some participants received a link to an online questionnaire on Facebook or e-mail while others gave their answers in a phone interview. The data material from 2016 was collected both at weekdays and weekends and the respondents were asked to specify which day they answered.

Ethical approval and research clearance was obtained from The National Committees for Research Ethics in Norway and from The Norwegian Social Science Data Services.

### Measures

At all time points, the questionnaire included the exact same questions concerning habitual beverage consumption, including “how often do you drink soda containing sugar?” and “how often do you drink diet soda?”. Both questions had ten response alternatives; never, less than once a week, once a week, twice a week, … six times a week, every day, several times a day. The response alternatives were later scored 0, 0.5, 1, 2 … 7, 10.

**Fig. 1:**
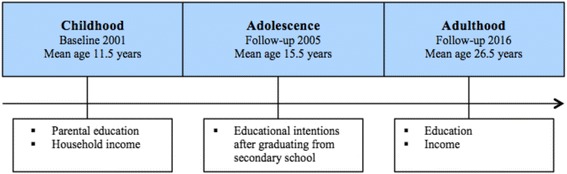
Indicators of socioeconomic status at different periods in life

Different measures of SES were used (Fig. 1). In 2001, the parents’ educational levels were assessed individually with one question: “what level of education do you have?” The question had four response alternatives: “elementary school”, “high school”, “college or university (three years or less)” and “college or university (more than three years)”. This variable was later dichotomized (lower: no college or university education, higher: having attended college or university). Household income was assessed by an open question on total household income. This variable was dichotomized into low or high household income with cut-off set at 450000 NOK (median).

In 2005, the participants were asked to indicate their plans for further education after graduating from secondary school. The response alternatives were “university or college”, “technical or vocational education”, “no further education” and “others”. This variable was dichotomized into lower (no plan to attend college or university) and higher (planning on attending college or university) educational intentions.

The participants’ own education was in 2016 assessed with the question “what level of education do you have?” The question had six response alternatives: “less than ten years of elementary school”, “elementary school”, “high school”, “college or university (four years or less)”, “college or university (more than four years), “others”. This variable was later dichotomized (lower: no college or university education, higher: having attended college or university). The participants’ income was assessed by an open question on total income and the variable was later dichotomized into low or high income with cut-off set at 350000 NOK (median).

### Statistical analyses

Statistical analyses were conducted using Statistical Package for Social Sciences, version 22 (IBM SPSS Statistics). Significance level were set at *p* ≤ 0.05.

Differences between the current sample and those lost to follow-up were analysed using a *t* test for continuous variables and chi-square statistics for the categorical variables (Table 1). For descriptive data, differences were analyzed using a *t* test and presented as mean (Table 2). The main analyses conducted were linear mixed effects analyses of the repeated measures (2001, 2005, 2016) with the SSB variable and ASB variable as outcome variables. All models included school as random effect, and time, gender, grade level and the different indicators of SES as fixed effects (one separate model for each SES indicator). In addition, second order interaction effects between time and the different indicators of SES were examined to assess development in socioeconomic inequality over time. The assumption for the linear mixed effects analysis of the repeated measures was met, as an examination of the residuals did not reveal unacceptable departures from normality.

**Table 1:** Baseline characteristics of the current study sample and those lost to follow-up

	Lost to follow-up	Current study sample	*p*-value^a^
Number	459	437	
Sex (% girls)	46.0	55.3	**0.007**
Parental education (% high)	30.0	47.3	**< 0.001**
Parental income (% high)	47.3	57.0	**0.026**
	Mean (SD)	Mean (SD)	*p*-value^b^
SSB (times/week)	2.7 ± (2.0)	2.8 ± (2.1)	0.631
ASB (times/week)	1.2 ± (1.8)	1.2 ± (1.7)	0.873

Statistical significant results at level *p* ≤ 0.05 are presented in bold

*SD* standard deviation

^a^ based on Chi Square test

^b^ based on independent samples *t*-test for continuous data

**Table 2:** Observed mean frequency for consumption of sugar-sweetened beverages and artificially sweetened beverages (times/week) at all time points

Year	2001	2005	2016	In total
	Mean	Mean	Mean	Mean
Sugar-sweetened beverages	2.8	2.4	1.2	2.1
Parental education 2001				
High	2.5	2.1	0.9	1.8
Low	2.9	2.7	1.3	2.3
*p*-value	0.090	**0.005**	**0.011**	**< 0.001**
Educational intentions 2005				
High	2.4	2.1	1.0	1.8
Low	3.1	2.9	1.5	2.5
*p*-value	**0.009**	**0.001**	**0.010**	**< 0.001**
Education 2016				
High	2.6	2.2	0.9	1.9
Low	3.2	3.0	1.9	2.7
*p*-value	**0.009**	**0.005**	**< 0.001**	**< 0.001**
Household income 2001				
High	2.6	2.3	1.0	1.9
Low	2.9	2.4	1.2	2.2
*p*-value	0.276	0.978	0.183	0.243
Income 2016				
High	2.8	2.4	1.0	2.1
Low	2.8	2.5	1.5	2.3
*p*-value	0.772	0.926	**0.012**	0.135
Artificially sweetened beverages	1.2	1.2	1.6	1.3
Parental education 2001				
High	1.0	1.0	1.4	1.3
Low	1.2	1.3	1.8	1.9
*p*-value	0.088	0.161	0.162	0.519
Educational intentions 2005				
High	1.0	1.1	1.5	1.2
Low	1.3	1.4	1.7	1.5
*p*-value	0.103	0.216	0.305	**0.028**
Education 2016				
High	1.2	1.2	1.5	1.3
Low	1.1	1.2	1.6	1.3
*p*-value	0.800	0.879	0.658	0.788
Household income 2001				
High	1.0	1.3	1.5	1.3
Low	1.2	1.1	1.6	1.3
*p*-value	0.340	0.513	0.683	0.696
Income 2016				
High	1.3	1.4	1.6	1.4
Low	1.1	1.0	1.5	1.2
*p*-value	0.525	0.058	0.688	0.109

Differences in beverage intake were analyzed using the independent-samples *t*-test

Statistical significant results at level *p* ≤ 0.05 are presented in bold

## Results

In total, 437 respondents were included in the study (49% of the baseline sample). Of the respondents 55.3% were girls, 47.3% had parents with higher education, and 57% had parents with higher income (Table 1). Compared to those lost to follow-up, the current study sample included significantly more girls and respondents with parents with higher education and income. No statistically significant differences were reported in baseline frequency of consumption of SSB (times/week) and ASB (times/week) between the current sample and those lost to follow-up.

Observed mean values for SSB (times/week) and ASB (times/week) frequency of consumption for all survey points are presented in Table 2. In general, the observed frequencies indicate a decrease in SSB frequency of consumption from 2001 (2.8 times/week) to 2005 (2.4 times/week) and a further reduction in 2016 (1.2 times/week).

Participants with higher educational levels in adulthood had a significantly lower SSB frequency of consumption at all time points (*p* = 0.009, *p* = 0.005, *p* = < 0.001) compared to those with a lower educational level. In addition, statistically significant differences between those planning to attend college or university and those with no plans for further education after graduating from secondary school were observed in 2001(*p* = 0.009), 2005 (*p* = 0.001) and 2016 (*p* = 0.010) where those with high SES had a lower frequency of consumption of SSB compared to those with low SES. Participants with parents with higher education had a significantly lower SSB frequency of consumption in 2005 (*p* = 0.005) and 2016 (*p* = 0.011) compared to those of parents with low education. Participants with low income in adulthood had a significantly higher frequency of consumption of SSB compared to those with high income in adulthood in the 2016 survey (*p* = 0.012), but not in 2001 and 2005. No statistically significant difference in SSB frequency of consumption was observed between high and low household incomes.

Observed mean frequency of ASB intake was 1.2 times/week in 2001 and 2005 compared to 1.6 times/week in 2016. ASB frequency of consumption at all time points was relatively stable in relation to all the SES indicators and no significant differences were observed between those with high and low SES for any of the SES indicators at any of the time points.

In this study, the linear mixed effects analysis of the repeated measures did not show any statistically significant interaction between time and the different socioeconomic indicators used for SSB or ASB (Table 3). I.e. there were no significant widening (or narrowing) of the relations between any SES indicator and SSB or ASB over time.

**Table 3:** Adjusted mean frequency for consumption of sugar-sweetened beverages and artificially sweetened beverages (times/week) according to time, gender, grade level and the different indicators of socioeconomic status

	2001		2005		2016		Interaction SES*TIME	In total		
	Mean	CI 95%	Mean	CI 95%	Mean	CI 95%	*p*-value	Mean	CI 95%	*p*-value^b^
Sugar-sweetened beverages	2.8	(2.5;3.0)	2.5	(2.3;2.8)	1.3	(1.1;1.5)	**< 0.001** ^**a**^			
Parental education 2001							0.389			**0.001**
High	2.6	(2.2;2.9)	2.1	(1.8;2.5)	1.0	(0.7;1.3)		1.9	(1.6;2.1)	
Low	3.0	(2.6;3.2)	2.8	(2.5;3.2)	1.4	(1.1;1.6)		2.4	(2.1;2.6)	
Difference	0.4		0.7		0.4			0.5		
Educational intentions 2005							0.580			**< 0.001**
High	2.5	(2.2;2.8)	2.2	(1.9;2.5)	1.0	(0.8;1.3)		1.9	(1.7;2.1)	
Low	3.0	(2.7;3.4)	2.8	(2.5;3.3)	1.5	(1.2;1.8)		2.5	(2.2;2.7)	
Difference	0.5		0.6		0.5			0.6		
Education 2016							0.148			**< 0.001**
High	2.7	(2.4;2.9)	2.3	(2.1;2.6)	1.0	(0.7;1.2)		2.0	(1.8;2.2)	
Low	3.1	(2.8;3.5)	2.9	(2.5;3.3)	1.9	(1.6;2.2)		2.7	(2.4;2.9)	
Difference	0.4		0.6		0.9			0.7		
Household income 2001							0.636			0.177
High	2.6	(2.3;3.0)	2.4	(2.1;2.8)	1.0	(0.7;1.3)		2.0	(1.7;2.3)	
Low	2.9	(2.5;3.3)	2.5	(2.1;2.8)	1.3	(1.0;1.6)		2.2	(2.0;2.5)	
Difference	0.3		0.1		0.3			0.2		
Income 2016							0.187			**0.038**
High	2.7	(2.4;3.0)	2.5	(2.1;2.8)	1.0	(0.7;1.3)		2.0	(1.8;2.3)	
Low	2.9	(2.6;3.2)	2.6	(2.2;2.9)	1.6	(1.3;1.9)		2.4	(2.1;2.6)	
Difference	0.2		0.1		0.6			0.4		
Artificially sweetened beverages	1.1	(1.0;1.3)	1.2	(1.0;1.4)	1.6	(1.4;1.8)	**0.002** ^a^			
Parental education 2001							0.054			0.553
High	0.9	(0.7;1.2)	1.1	(0.8;1.4)	1.8	(1.4;2.1)		1.3	(1.1;1.5)	
Low	1.3	(1.0;1.5)	1.3	(1.0;1.6)	1.5	(1.1;1.8)		1.3	(1.1;1.5)	
Difference	0.4		0.2		0.3			0.0		
Educational intentions 2005							0.996			**0.038**
High	1.0	(0.8;1.2)	1.1	(0.9;1.4)	1.4	(1.2;1.7)		1.2	(1.0;1.4)	
Low	1.3	(1.0;1.6)	1.4	(1.1;1.7)	1.7	(1.4;2.1)		1.5	(1.3;1.7)	
Difference	0.3		0.3		0.3			0.3		
Education 2016							0.799			0.972
High	1.2	(1.0;1.4)	1.2	(1.0;1.5)	1.5	(1.3;1.8)		1.3	(1.1;1.5)	
Low	1.1	(0.8;1.4)	1.2	(0.8;1.5)	1.6	(1.3;2.0)		1.3	(1.1;1.5)	
Difference	0.1		0.0		0.1			0.0		
Household income 2001							0.342			0.872
High	1.0	(0.7;1.2)	1.3	(0.1;1.6)	1.5	(1.2;1.8)		1.3	(1.1;1.5)	
Low	1.2	(0.9;1.5)	1.1	(0.7;1.4)	1.6	(1.2;2.0)		1.3	(1.0;1.5)	
Difference	0.2		0.2		0.1			0.0		
Income 2016							0.671			0.216
High	1.3	(1.0;1.5)	1.4	(1.1;1.7)	1.7	(1.3;2.0)		1.4	(1.2;1.6)	
Low	1.2	(0.9;1.4)	1.1	(0.8;1.4)	1.5	(1.2;1.9)		1.3	(1.0;1.5)	
Difference	0.1		0.3		0.2			0.1		

^**a**^p-value TIME

^**b**^p-value SES indicator

Multilevel repeated measures adjusted for time, gender, grade level and different indicators of socioeconomic status (one separate model for each indicator)

Statistical significant results at level *p* ≤ 0.05 are presented in bold

## Discussion

From 2001 to 2016 there has been a decrease in frequency of consumption of SSB and an increase in frequency of consumption of ASB. At all three time points (2001, 2005 and 2016), participants with low SES reported higher frequency of consumption of SSB than those with high SES when educational intentions in adolescence and participants’ own education in adulthood were used as indicators of SES. The results from the present study showed no significant widening in the social inequalities in frequency of consumption of SSB and ASB from childhood to adulthood.

In agreement with sales figures in Norway, the present study confirms the decrease in consumption of SSB [17]. The last years’ increased focus on structural initiatives, such as fiscal policies, to reduce intake of added sugars and consumption of SSB in the Norwegian population may have affected the observed decrease in frequency of consumption of SSB in the present study [10–13]. This decrease is also reported among Norwegian children from 2001 and 2008 [34].

In comparison with international trends, the present study shows an increased frequency of consumption of ASB in the last years [24]. It is reasonable to assume that the observed increased trend in frequency of consumption of ASB is partly due to greater accessibility and selection of ASB in addition to the promotion of ASB as a healthier alternative to SSB [35]. For instance, in 2007 a report from the Norwegian Scientific Committee for Food Safety concluded that ASB should be recommended as a healthier alternative to SSB as replacing sugar with intense sweeteners in soft drinks may reduce the risk of weight gain [36].

Our results regarding SES inequalities in SSB correspond with earlier studies showing that adolescents without higher education intentions had greater odds for drinking SSB more often than those planning to attend college or university and that adults with a lower educational level have a higher intake of SSB in relation to those with a higher educational level [19, 22]. In contrast to our results, earlier research suggests an inverse association between consumption of ASB and education, where those with a higher educational level have an increased consumption of ASB compared to those with a lower educational level [26]. The observed increasing trend in consumption of ASB in the present study emphasizes the need for further investigation into the relation between ASB and SES as few studies have established a social gradient for the consumption of ASB.

It has been argued that the relative low prices on SSB could be an explanation as to why a lower SES is associated with a higher consumption of SSB [37]. In the present study, both parental household incomes measured in 2001 and participants’ own income in 2016 show negligible differences. Income is likely to mirror the availability of economic and material resources, and therefore influence dietary quality by making healthy food more or less affordable and accessible. With respect to healthy food choices, it seems that economic factors are not enough for healthy eating [38].

Studying social inequality in diet among adolescents is often a question about the extent to which parents and home conditions affect diet [39]. Our study indicates that adolescents’ educational intentions relate closely to frequency of consumption of SSB in both adolescence and adulthood. This result is supported by results from another study which showed that educational intentions at age 13 and 15 were stable and tracked well into adulthood [40]. This might imply that adolescents’ personal SES should be included in the studies of health inequality, a finding that is also supported by others [40–42].

The present study showed no significant change in socioeconomic inequality in frequency of consumption of SSB and ASB from childhood to adulthood in the period from 2001 to 2016. As reducing socioeconomic inequality in health and health-related behaviors is a primary goal of public health policy in Norway, these results are encouraging. Usually, improvements in general population diet are due to improvements in the diet of those with high SES as this group is more likely than low SES groups to respond to dietary recommendations [43], something which results in increasing inequalities. Currently, high social inequalities in health persist in the Nordic countries, described as the Nordic paradox [44]. Our results may indicate that the structural efforts (increased tax on non-alcoholic beverages, reduced food marketing to children, school meal recommendations) conducted in Norway may have counteracted a trend of increasing social inequalities. Such structural efforts have been reported to have this effect of potentially reducing social inequalities, compared to efforts more targeted to individual change decision making [45, 46].

Studies investigating socioeconomic differences in diet are often very descriptive in nature, and tend to not seek to understand the underlying construct of the observed relationships [47]. A major strength of the present study is the use of a longitudinal design, following a sample from childhood to adulthood, using different indicators of SES measured at different stages in life, in order to better understand how these indicators are related to soft drink consumption.

The results presented in this paper should also be seen in relation to our research’s limitations. Firstly, a limitation to the study is its relatively small sample size. The pupils in the 20 control schools constitute the study sample of the present study and the 18 intervention schools were excluded. By not including all the schools, statistical power is reduced. However, sensitivity analysis conducted including the full sample revealed the same results. Based on these analyses, a decision was made to only include control data and reduce the risk of confounding due to the study intervention. Secondly, the variables of SSB and ASB consumption frequency have not been validated. However, based on data from the previous test-retest study involving 114 children of 6th grade, individual scores were significantly (*p* <  0.001) correlated (Person’s correlation coefficient) for SSB (*r* = 0.72) and ASB (*r* = 0.44), which indicated an acceptable level of reproducibility [34, 48]. Thirdly, the final study sample was more likely to have highly educated parents and parents with high income than those not participating in the last survey (attrition). As parental education and income are assumed to be determinants of children’s and adolescents’ SSB and ASB consumption, the differences between socioeconomic groups may have been underestimated [25, 49, 50]. Further, the questionnaire did not have information about volume obtained from beverages, and beverage consumption has been reported only three times across a 15 years period and may not fully represent typical consumption patterns across this time frame. Lastly, the participants were from two of Norway’s 19 counties only. However, Norway is a rather homogenous country and the results may probably be generalized to all parts of Norway.

## Conclusions

A decrease in consumption of SSB and an increase in consumption of ASB from childhood to adulthood were found. Participants with high SES consumed in general less SSB (but not ASB), however results varied depending on SES indicators used. The established inequalities persisted from childhood to adulthood.

## Abbreviations

ASB: Artificially sweetened beverages
FVMM: The Fruits and Vegetables Make the Marks-project
SES: Socioeconomic status
SSB: Sugar-sweetened beverages

## References

[1] Arcaya MC, Arcaya AL, Subramanian SV. Inequalities in health: definitions, concepts, and theories. Glob Health Action. 2015;8:27106. 10.3402/gha.v8.27106 PMC4481045 26112142

[2] Marmot M, Bell R (2016). Social inequalities in health: a proper concern of epidemiology. Ann Epidemiol.

[3] Mackenbach JP (2006). Health inequalities: Europe in profile.

[4] Fismen AS, Smith OR, Torsheim T, Rasmussen M, Pedersen Pagh T, Augustine L, Ojala K, Samdal O (2016). Trends in food habits and their relation to socioeconomic status among Nordic adolescents 2001/2002-2009/2010. PLoS One.

[5] Dahl E, Bergsli H, van der Wel KA (2014). Sosial ulikhet i helse: En norsk kunnskapsoversikt.

[6] Meld. St. 19 (2014–2015): Folkehelsemeldingen- mestring og muligheter Oslo: Helse- og Omsorgsdepartementet; 2015.

[7] de Gelder R, Menvielle G, Costa G, Kovacs K, Martikainen P, Strand BH, Mackenbach JP (2017). Long-term trends of inequalities in mortality in 6 European countries. Int J Public Health.

[8] Ministry of Health and Care Services (2007). Norwegian action plan on nutrition (2007–2011) - «recipe for å healthier diet».

[9] Ministry of Health and Care Services (2017). Norwegian action plan on nutrition (2017–2021) - «a healthy diet and good health for all».

[10] The Norwegian Directorate of Taxes (2016). Taxes on non-alcoholic beverages mv 2016.

[11] The Norwegian Directorate of Customs and Excise (2008). Taxes on non-alcoholic beverages 2008, circular letter 4/2008.

[12] The Norwegian Directorate of Customs and Excise (2010). Taxes on non-alcoholic beverages 2010, circular letter 4/2010.

[13] The Norwegian Consumer Council. Marketing guidelines for foods an beverages to children og young people. Oslo: The Norwegian Consumer Council; 2007.

[14] World Health Assembly agenda item A63/12. Includes Annex: set of recommendations on the marketing of food and non-alcoholic beverages to children. [http://apps.who.int/gb/ebwha/pdf_files/WHA63/A63_12-en.pdf]. Accessed 12 Jan 2018.

[15] The Norwegian Directorate of Health, Health TNDo (2015). National guidelines regarding food and meals in school.

[16] The Norwegian Directorate of Health (2014). Norwegian recommendations on nutrition, diet and physical activity.

[17] The Norwegian Directorate of Health (2016). Developments in the Norwegian diet 2016.

[18] Hansen LB, Myhre JB, Johansen AMW, Paulsen MM, Andersen LF (2016). UNGKOST 3, Nationwide dietary survey among pupils in 4th and 8th grade in Norway, 2015.

[19] Totland TH, Melnæs BK, Lundeberg-Hallén N, Helland-Kigen KM, Blix NAL, Myhre JB, Johansen AMW, Løken EB, Andersen LF (2012). Norkost 3 - a nationwide food consumption survey among men and woman in Norway age 18–70, 2010–2011.

[20] Kvaavik E, Andersen LF, Klepp KI (2005). The stability of soft drinks intake from adolescence to adult age and the association between long-term consumption of soft drinks and lifestyle factors and body weight. Public Health Nutr.

[21] Samdal O, Mathisen F, Torsheim T, Diseth Å, Fismen AS, Larsen T, Wold B, Årdal E: Helse og trivsel blant barn og unge. Resultater fra den landsrepresentative spørreundersøkelsen «Helsevaner blant skoleelever. En WHO-undersøkelse i flere land». vol. HEMIL-Rapport 1/2016. Bergen: HEMIL-senteret: Universitetet i Bergen; 2016.

[22] Bere E, Glomnes ES, te Velde SJ, Klepp KI (2008). Determinants of adolescents’ soft drink consumption. Public Health Nutr.

[23] Paulsen MM, Myhre JB, Andersen LF. Beverage consumption patterns among Norwegian adults. Nutrients. 2016;8. 10.3390/nu8090561. 10.3390/nu8090561 PMC5037546 27649236

[24] Sylvetsky AC, Rother KI (2016). Trends in the consumption of low-calorie sweeteners. Physiol Behav.

[25] Pereira MA (2013). Diet beverages and the risk of obesity, diabetes, and cardiovascular disease: a review of the evidence. Nutr Rev.

[26] Drewnowski A, Rehm CD (2015). Socio-demographic correlates and trends in low-calorie sweetener use among adults in the United States from 1999 to 2008. Eur J Clin Nutr.

[27] Adler NE, Ostrove JM (1999). Socioeconomic status and health: what we know and what we Don't. Ann N Y Acad Sci.

[28] Bere E, Klepp KI. Free Fruit for School Children to Improve Food Quality. In: Preedy V., Hunter LA., Patel V. (eds) Diet Quality. New York: Nutrition and Health. Humana Press; 2013.

[29] Bere E, Klepp KI, Overby NC. Free school fruit: can an extra piece of fruit every school day contribute to the prevention of future weight gain? A cluster randomized trial. Food Nutr Res. 2014;58. 10.3402/fnr.v58.23194. 10.3402/fnr.v58.23194 PMC4131001 25147495

[30] Bere E, te Velde SJ, Smastuen MC, Twisk J, Klepp KI (2015). One year of free school fruit in Norway--7 years of follow-up. Int J Behav Nutr Phys Act.

[31] Bere E, van Lenthe F, Klepp KI, Brug J (2008). Why do parents’ education level and income affect the amount of fruits and vegetables adolescents eat?. Eur J Pub Health.

[32] Bere E, Veierod MB, Klepp KI (2005). The Norwegian school fruit Programme: evaluating paid vs. no-cost subscriptions. Prev Med.

[33] Øverby NC, Klepp KI, Bere E (2012). Introduction of a school fruit program is associated with reduced frequency of consumption of unhealthy snacks. Am J Clin Nutr.

[34] Stea TH, Overby NC, Klepp KI, Bere E (2012). Changes in beverage consumption in Norwegian children from 2001 to 2008. Public Health Nutr.

[35] Borges MC, Louzada ML, de Sa TH, Laverty AA, Parra DC, Garzillo JM, Monteiro CA, Millett C (2017). Artificially sweetened beverages and the response to the global obesity crisis. PLoS Med.

[36] Andersen LF, Husøy T, Kolset SV, Jakobsen HN (2007). Impact on health when sugar is replaced with intense sweeteners in soft drinks, ‘saft’ and nectar.

[37] Jensen JD, Bere E, De Bourdeaudhuij I, Jan N, Maes L, Manios Y, Martens MK, Molnar D, Moreno LA, Singh AS (2012). Micro-level economic factors and incentives in Children's energy balance related behaviours - findings from the ENERGY European cross-section questionnaire survey. Int J Behav Nutr Phys Act.

[38] Skardal M, Western IM, Ask AM, Overby NC. Socioeconomic differences in selected dietary habits among Norwegian 13-14 year-olds: a cross-sectional study. Food Nutr Res. 2014;58. 10.3402/fnr.v58.23590 PMC4111874 25140123

[39] Elgar FJ, McKinnon B, Torsheim T, Schnohr CW, Mazur J, Cavallo F, Currie C (2016). Patterns of socioeconomic inequality in adolescent health differ according to the measure of socioeconomic position. Soc Indic Res.

[40] Friestad C, Lien N, Klepp KI (2001). Educational plans - when are they estabished? Implications for the measurement of socioeconomic status in youth. Young.

[41] Koivusilta LK, Rimpela AH, Kautiainen SM (2006). Health inequality in adolescence. Does stratification occur by familial social background, family affluence, or personal social position?. BMC Public Health.

[42] Hilsen M, te Velde SJ, Bere E, Brug J (2013). Predictors and mediators of differences in soft drinks consumption according to gender and plans of further education among Norwegian secondary-school children. Public Health Nutr.

[43] Matthiessen J, Andersen LF, Barbieri HE, Borodulin K (2016). The Nordic Monotoring system 2011–2014: status and development of diet, physical activity, smoking, alcohol and overweight.

[44] Mackenbach JP (2017). Nordic paradox, southern miracle, eastern disaster: persistence of inequalities in mortality in Europe. Eur J Pub Health.

[45] Lorenc T, Petticrew M, Welch V, Tugwell P (2013). What types of interventions generate inequalities? Evidence from systematic reviews. J Epidemiol Community Health.

[46] McGill R, Anwar E, Orton L, Bromley H, Lloyd-Williams F, O’Flaherty M, Taylor-Robinson D, Guzman-Castillo M, Gillespie D, Moreira P (2015). Are interventions to promote healthy eating equally effective for all? Systematic review of socioeconomic inequalities in impact. BMC Public Health.

[47] Braveman PA, Cubbin C, Egerter S, Chideya S, Marchi KS, Metzler M, Posner S (2005). Socioeconomic status in health research: one size does not fit all. JAMA.

[48] Andersen LF, Bere E, Kolbjornsen N, Klepp KI (2004). Validity and reproducibility of self-reported intake of fruit and vegetable among 6th graders. Eur J Clin Nutr.

[49] Han E, Powell LM (2013). Consumption patterns of sugar-sweetened beverages in the United States. J Acad Nutr Diet.

[50] Fakhouri TH, Kit BK, Ogden CL (2012). Consumption of diet drinks in the United States, 20092010. NCHS Data Brief.

